# Pneumonia and Empyema at Camp Zachary Taylor, Ky.

**Published:** 1918-11

**Authors:** 


***Pneumonia and Empyema at Camp Zachary Taylor, Ky.*** By WALTER W. HAMBURGER and LAWRENCE H. MAYERS. *Journal of the American Medical Association,* March 30, 1918.

The early cases in September and October apparently conformed to the usual picture of lobar pneumonia, with sudden onset, chill, temperature, rusty sputum, localized areas of consolidation, etc. These pneumonias undoubtedly belong to the more benign type of organisms, as only one death occurred in the series.

The first case definitely diagnosed broncho-pneumonia occurred ten days after the first measles admission. From then on the number of cases of pneumonia following measles increased rapidly, paralleling closely the measles admissions, latterly decreasing *pari passu.* They were most severe and fulminant. Developing usually during the third or fourth day of the rash, pneumonic process was ushered in by an increase in temperature, respiratory rate, patchy areas of dullness, roughened broncho-vesicular breathing, moist bubbling râles, and, of particular interest, marked dyspnea, and dusky cyanosis of the face. The two latter symptoms were most distressing and difficult to relieve.

From an epidemiological standpoint, considerable interest is attached to the time of development of the pneumonia in relation to the period of hospitalization or discharge to quarters. The majority of cases developed while in the hospital wards, as close sequelae to the primary disease, either as contact or droplet infections from other patients, ward air or ward dust, or from the patients' own bacterial flora from mouth or nasopharynx, the minority developing after a return to duty.

About December 1st, the fulminant atypical lobar pneumonias, later proved to be streptococcal in origin, began to appear. From the standpoint of the prodromes, these cases divide themselves into two classes:

1. Those starting insidiously with sore throat, cough, and grippe, for a few days, progressing gradually until frank signs of consolidation could be elicited.

2. Those starting abruptly, severely, with sudden overwhelming prostrations and collapse, profoundly toxic, progressing rapidly to death in three or four days, with symptoms so profound and acute as to suggest a general sepsis. Digitalis, caffein, venesection — nothing seemed to make the slightest impression.

In both of these groups of cases, empyema developed in an extremely high percentage. The variations in the empyema curve were quite separate and distinct from the pneumonic curve per se, and were probably due to the virulence of the invading organisms.,

Many cases, diagnosed clinically massive lobar pneumonia, showed at necropsy widespread atelectasis with little or no evidence of pneumonic consolidation. Purulent pericarditis was frequently associated, the pericardeum containing a pint or more of thick, creamy pus.

Hemolytic streptococci were found in pure culture in a majority of the empyema fluids. The severity and rapidity of the onset, the overwhelming prostration and toxemia, metastatic abscesses in joints, cellular tissues, pericardeum, middle ear, mastoid, meninges and brain, and the inefficacy of treatment, all lend strong support to the view that these cases are true examples of streptococcus sepsis.

The routine of examination and handling of these patients is as follows:

1. Every patient showing broncho-pneumonia and lobar pneumonia, severe sepsis, continued high temperature, severe continued dyspnea, and a tightness in the chest, should be considered as having potential empyema.

2. Each potential empyema patient should be roentgenographed within 48 hours after admission.

3. Each potential empyema patient should be seen within 48 hours after admission for consultation regarding exploratory thoracentesis.

4. A positive thoracentesis should be followed immediately by the Potain treatment, as much fluid being withdrawn as is compatible with the patient's condition.

5. On the fluid's becoming opaque and of creamy consistency, the patient should be transferred to the surgical service for continuous drainage with suction apparatus.

The use of intercostal drainage, with the attached water pump for continuous suction, has been superior to other forms of surgical treatment. This method imposes a minimum of surgical trauma, prevents the aspiration of foreign material and collapse of the lung from pneumothorax, allows the pus to escape gradually, maintains negative interpleural pressure, and shortens the patient's period of invalidism.

